# Consumption

**Published:** 1898-10

**Authors:** C. O. Probst

**Affiliations:** Secretary; Columbus, O.


### Consumption.

Ohio State Board of Health, Office of Secretary. Columbus, O.

*To the Editor:*

Dear Sir: I send you enclosed a circular just issued by the State Board of Health on "The Prevention of Consumption,"

and earnestly request your assistance in making public the information it contains.

There is no longer a doubt as to the contagiousness of consumption, nor that the disease may, to a large extent, be prevented. For many reasons it is impracticable, if not undesirable, to treat consumptive patients as we do those afflicted with diphtheria, small pox, or contagious diseases of that class. A consumptive is not dangerous to others with proper care, and at present we must depend on an educational campaign.

More deaths (from consumption) occur each year in Ohio than from all the other contagious diseases combined; and with your help we hope to impress the general public with the fact that this great annual slaughter can be very materially lessened.

I shall be greatly obliged if you will kindly send me a copy of what you publish on this subject. Respectfully,

C. O. PROBST, M. D., Secretary.

---

*To the Superintendent of Schools:*

DEAR SIR: The State Board of Health has determined to commence a crusade against consumption. It has been decided that for the present this must be largely a matter of education. A circular* on "The Prevention of Consumption," enclosed herewith, was prepared for popular instruction.

The physicians of Ohio have agreed to place copies of this circular in all families where they are called on account of consumption. The circular will also be printed by a large number of our newspapers; and we hope to enlist your aid in spreading the information this circular contains.

There is no longer a doubt that consumption may be communicated from one person to another. Hundreds of such cases have been reported to us by the physicians of Ohio. It is also true that this may be often prevented by measures easily enforced.

We desire to know whether you think it would be advisable to impart such information to advanced pupils under your charge. It would doubtless be ill-advised to excite unnecessary alarm, and especially to too seriously impress children with the danger of associating with a consumptive person. But would it not be well that every pupil leaving school should realize that

[*No copy of circular mentioned was enclosed or since received.—ED.]

one-seventh of all who die are victims to a disease which in many cases might be prevented? That this disease may be communicated by the sick to the well, but that this may be prevented by comparatively simple measures.

Let our people fully realize the dangers that encompass them and how these dangers may be averted, and we may confidently expect that the law of self-preservation will work out the remedy.

Additional copies of the circular on "Prevention of Consumption" will be sent you if desired. Respectfully,

C. O. PROBST, M. D., Secretary.

